# Trends and determinants of minimum acceptable diet intake among infant and young children aged 6–23 months in Ethiopia: a multilevel analysis of Ethiopian demographic and health survey

**DOI:** 10.1186/s40795-022-00533-8

**Published:** 2022-05-05

**Authors:** Firanbon Teshome, Afework Tadele

**Affiliations:** 1 Department of Health, Behavior and Society, Faculty of Public Health, Jimma University, Jimma, Ethiopia; 2 Department of Population and Family Health, Faculty of Public Health, Jimma University, Jimma, Ethiopia

**Keywords:** Minimum acceptable diet, Infant and young children, Ethiopia

## Abstract

**Background:**

The minimum acceptable diet (MAD) has been used globally as one of the main indicators to assess the adequacy of feeding practices. More than half of the causes of under-five child mortality in developing countries including Ethiopia are attributed to malnutrition. With the exception of anecdotal information on the subject, progress overtime and how it influences the MAD has not been studied or well understood. Thus, this study aimed to determine the trends and determinants of MAD intake among infants and young children aged 6–23 months in Ethiopia.

**Methods:**

A community-based national survey dataset from the Ethiopian demographic and health survey (EDHS) 2019 were to identify predictors of MAD. In addition, the 2011, 2016, and 2019 EDHS data was used for trend analysis. The World Health Organization indicators were used to measure MAD. A weighted sample of 1457 infants and young children aged 6–23 months. A mixed-effects multi-level logistic regression model was employed using STATA version 16.0.

**Results:**

The proportions of infants and young children who received the MADs in Ethiopia were 4.1%, 7.3%, and 11.3% during the survey periods of 2011, 2016, and 2019, respectively. Having mothers who attended primary education [adjusted odds ratio (aOR) =2.33 (95% C.I 1.25 to 4.35)], secondary education [aOR = 2.49 (95% C.I 1.03 to 6.45)], or higher education [aOR = 4.02 (95% C.I 1.53 to 10.54)] compared to those who never attended formal education. Being in a medium househoold wealth [aOR = 4.06 (95% C.I 1.41 to 11.72)], higher-level wealth [aOR = 4.91 (95% C.I 1.49 to 16.13)] compared to those in the lowest househoold wealth. Being in 12–18 months age group [aOR = 2.12 (95% C.I 1.25 to 3.58)] and in 18–23 months age category [aOR = 2.23 (1.29 to 3.82)] compared to 6–11 months age group; and having postnatal check-ups [aOR = 2.16 (95% C.I 1.31 to 3.55)] compared to their counterparts. Moreover, residing in urban [aOR = 3.40 (95% C.I 1.73 to 6.68)]; living in a communities’ where majority had a media exposure [aOR 1.80 (95% C.1.17 to 2.77)] were found to be significantly influenc consumption of the MAD.

**Conclusions:**

The trends of MAD among children of 6–23 months was steady in Ethiopia. Sociodemographic and socioeconomic factors such as maternal education, child age, household wealth; and health system related factors such as maternal postnatal check-ups had a significant influence on infants’ and young children’s MAD feeding. Indeed, commnity-level factors such as place of residence, and media exposure affect the MAD of infants and young children. Thus, behavioral change communication interventions are recommended to improve dietary practices in infants and young children.

## Introduction

Globally, in 2013 approximately 6.3 million under-five age children died and among those approximately 46% of all children, death is expected to occur in the sub-Saharan Africa region [1–3]. Worldwide, malnutrition is directly or indirectly responsible for 60% of under5-year deaths and two-thirds of the deaths are associated with inappropriate feeding practices [4]. In developing countries, under-nutrition accounts for 45% of under-five child mortality [5]. In Ethiopia national rates of wasting, underweight, stunted among under 5 years children were 7, 21, and 37%, respectively. This may be mainly due to the low minimum acceptable diet in the county where only 11.3% of children met the requirement [6].

The minimum acceptable diet is the proportion of children aged 6–23 months who receive a minimum diversified diet and minimum meal frequency [7]. Dietary diversity is defined as the number of individual food items or groups consumed over a reference period, and has been accepted as an aspect of dietary quality and can indicate nutritional adequacy [8]. It is the proportion of children 6–23 months of age who received at least 4 of seven recommended food groups daily, including grains, roots and tubers, legumes and nuts, dairy products (milk, yogurt, cheese), flesh foods (meat, fish, poultry and organ meats), eggs, vitamin-A rich fruits and vegetables and other fruits and vegetables [9].

Minimum acceptable diet is one of the eight core indicators of complementary feeding. It is an important and cost-effective strategy during the first two years of life to improve survival, growth, and development, reduce morbidities, mortalities of young children; prevent chronic degenerative disease; and improve health in later life [10–12]. It is one of the pillars of public health interventions worldwide [13]. The Minimum acceptable diet has been used globally as one of the main indicators for assessing the adequacy of feeding practices [14]. However, less than a quarter of children aged 6–23 months get the recommended diversified diet globally [15].

Infant and young child feeding practices are a global challenge especially in developing countries where their diets are predominantly based on starchy foods with little or no animal products and few fresh fruits and vegetables [16]. Poor infant and young child feeding practices during the first two years of life have both short-term consequences such as impaired cognitive development, growth retardation, stunting, inadequate immune response, compromised educational achievement, increased risk of childhood morbidities, mortalities, disabilities, poor reproductive performance, low economic productivity and intergenerational consequences [17, 18]. With all these problems, the world is far from attaining sustainable development goal 3 which aims to end preventable deaths of new-borns and children under five years by 2030 [19].

Although efforts have been made, Ethiopia has ranked lowest in East African countries regarding infant and young child feeding practices [20]. Dietary diversity has been extensively studied in Ethiopia [21–24]. However, dietary diversity alone may not be sufficient to indicate appropriate infant and young child feeding practices and to improve the health of infants and children. Hence, considering both the diversified diet and frequency of meals, and attaining the daily recommended minimum acceptable diet has paramount importance. Despite its importance and recommendation [10–12], there is a paucity of studies on the minimum acceptable diet, its progress overtime and predicting factors. Therefore, this study aimed to determine the trends and determinants of a minimum acceptable diet among infants and young children aged 6–23 months in Ethiopia, which will help policymakers, program planners, and local managers to design feasible interventions.

## Methods

### Data source

The study utilized the Ethiopian Demographic Health Survey (EDHS) datasets obtained from the Demographic Health Survey (DHS) program website http://dhsprogram.com/data/ in the STATA format. The survey covered all the nine regions and two city administrations of Ethiopia. In this study, the 2011, 2016, and 2019 EDHS data were used for trend analysis. Accordingly, data were collected from 2949 children aged 6–23 months for EDHS 2011, 2919 for EDHS 2016, and 1457 for EDHS 2019 and trend analysis was carried out. To identify the predictors of MAD, data from the EDHS 2019 were utilized.

### Tool, definitions of study variables, and measurements

In this study, the World Health Organization 2008 indicators for Assessing Infant and Young Child Feeding were used to measure MAD.

#### Outcome variable

**Fig. 1:**
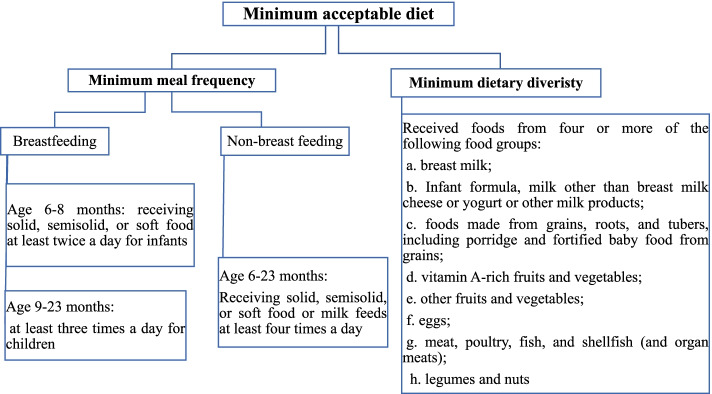
Diagrammatic presentation of minimum acceptable diet among 6–23 months aged children in Ethiopia

Is the minimum acceptable diet which is a composite index of minimum dietary diversity and minimum meal frequency. Children aged 6–23 months were considered to be fed a minimum acceptable diet (coded as 1 otherwise coded as 0) if they received breast milk, other milk, or milk products; were fed the minimum dietary diversity, and were fed the minimum meal frequency [25] (Fig. 1).

#### Minimum dietary diversity

The proportion of children aged 6–23 months who received foods from four or more of the seven food groups [25].

#### Minimum meal frequency

Proportion of breastfed and non-breastfed children aged 6–23 months who received solid, semisolid, or soft foods (but also including milk feeds for non-breastfed children) with the minimum number of 2 and 3 times for those breastfed infants and children aged 6–23 months and 4 times for non-breastfed infants and children aged 6–23 months, respectively [25].

#### Explanatory variables

The explanatory variables for minimum dietary diversity were broadly classified into individual and community-level factors in line with a multilevel analytic approach.

#### Individual-level factors

Included age of children, women’s education, women’s age, household wealth index, birth order, ANC visits, place of delivery, postnatal check within 2 months, counselling on danger signs, and counselling on breastfeeding.

#### Community-level factors

Are the physical and social surroundings of individuals and households or families that either increase or decrease an individual’s likelihood of engaging in a certain behavior. In this study the wealth index, women’s education, media exposure, place of residence, ANC utilization rate, and region were community-level factors.

#### Community-level women’s education

The median value of educational attainment at the national level was 5 years. Thus, the median value of the aggregated clusters below 5 was classified as low education for women and the median value of the aggregated clusters 5 and more were classified as high education for women [6].

#### Community-level wealth index

Refers to community poverty, computed based on the median values of individual household wealth categories. The aggregated clusters were then classified as low or high based on the median value of the clusters.

### Residence

This variable in the EDHS directly explains the cluster characteristics. The two categories are: urban, and rural.

### Community media exposure

A woman was said to have media exposure if at least exposed to one of the three sources of information (TV, radio, or newspaper), otherwise not. If greater than or equal to 50% of the cluster members exposed to media, we considered as having high community-level media exposure and otherwise low.

### Region

The Ethiopian administrative nine regional states and two city administrations were classified into three groups (agrarian, pastoralists, and city) based on their settings which may have a strong relationship with health-seeking behaviours. Six regions (Tigray, Amhara, Oromiya, SNNP, Gambella, and Benshangul Gumuz) were recorded as *“agrarian regions”*. Two regions (Somali and Afar) were combined to form *“pastoralist regions”* and the city administrations (Addis Ababa, Dire Dawa, and Harar) were categorized as *“city”* [26].

### Data analysis

Two-level mixed-effects logistic regression analyses were conducted using STATA version 16.0 with the xtmelogit command [27]. The 2019 mini-EDHS dataset was considered as hierarchical, by first-level individuals (6–23 months children) nested in households, and the households were nested in the cluster. The unit of analysis for the characteristics of community-level factors was the cluster. For this study, we included 296 clusters in which all the 6–23 months children within two years preceding the survey reside.

Bi-variable two-level mixed-effects logistic regression analyses were employed to identify the association between the outcome and explanatory factors of the study. The overall categorical variables with a *p*-value of < 0.25 in the bivariate two-level mixed-effect logistic regression analysis were included in the final model of the multivariable two-level mixed-effect logistic regression model in which odds ratios with 95% confidence intervals were estimated to identify the explanatory variables of children aged 6–23 months receiving minimum acceptable diet. Statistical significance was set at *p* < 0.05. All analyses were performed on weighted data using survey data analysis to avoid problems that could arise from the non-proportional distribution of clusters by region and to make the regional distribution nationally representative.

In this analysis four models were fitted: *Model I (Empty model)* was fitted without explanatory variables to test random variability in the intercept and to estimate the intraclass correlation coefficient (ICC). *Model II* examined the effects of individual-level characteristics; *Model III* examined the effect of community-level variables and *Model IV* examined the effects of both individual and community-level characteristics simultaneously [28].

The intra-class correlation (ICC) was calculated using the STATA post-estimation command estat icc to show the proportion of the between cluster variation in the total variation. The variability in the odds of receiving the minimum acceptable diet explained by successive models was calculated using the Proportional Change in Variance (PCV) [27]. As shown in the null model, 23.3% of the variation in receiving the minimum acceptable diet could be attributed to community characteristics. The log-likelihood ratio was used to fit the models [29] (Table 3).

## Results

### Individual-level characteristics of study participants

Findings showed that 550(37.74%) of children were in the age range of age 12–18 months and of them, only 77 (5.28%) received the minimum acceptable diet. Among the 645 women who had no formal education, only 45(3.09) of their children received the minimum acceptable diet. Of the total of 1457 participants, the majority 1259(86.41%) hadn’t got a postnatal check-up within two months after delivery. One-third, 486(33.36%) of individuals who had medium wealth didn’t get minimum acceptable diet (Table 1).

**Table 1:** Distribution of background and obstetric characteristics of individual-level factors, analysis from the 2019 EDHS, (weighted *N* = 1457)

Individual-level variables	Minimum acceptable diet		Total (weighted) N (%)
	Acceptable, N (%)	Not acceptable, N (%)	
Age of children			
6–11 months	30 (2.06)	446 (30.61)	476 (32.67)
12–18 months	77 (5.28)	473 (32.46)	550 (37.74)
18–24 months	56 (3.84)	374 (25.67)	430 (29.51)
Highest education			
No education	45 (3.09)	600 (41.18)	645 (44.27)
Primary	80 (5.49)	529 (36.31)	609 (41.80)
Secondary	23 (1.58)	97 (6.66)	120 (8.24)
Higher	16 (1.10)	66 (4.53)	82 (5.63)
Wealth			
lowest	25 (1.72)	421 (28.89)	446 (30.61)
medium	69 (4.74)	486 (33.36)	555 (38.09)
Highest	70 (4.80)	386 (26.49)	456 (31.29)
Place of delivery			
Home	53 (3.64)	578 (39.67)	631 (43.31)
Institution	112 (7.69)	714 (49.00)	826 (56.69)
Postnatal check within 2 months			
No	124 (8.51)	1135 (77.90)	1259 (86.41)
Yes	41 (2.81)	157 (10.78)	198 (13.59)

### Community-level characteristics of study participants

The study included 305 clusters, in which all children in the age group of 6 to 23 months lived. The Majority, 980(67.26%) and 1044(71.65%) of clusters had a lower proportion of the community level women education, and rural residents, respectively. The findings showed that only 102 (7.00%) of children in rural received the minimum acceptable diet. The majority, (88.33%) of clusters were agrarian regions. Of these, only 10.09% of children got a minimum acceptable diet. More than half, 54.77% of clusters had a lower proportion of community antenatal care, with only 5.15% of children who got minimum acceptable diet. The finding indicated that three fourth, (74.81%) of clusters had a lower proportion of community media access. Of these, only 7.00% of children received a minimum acceptable diet (Table 2).

**Table 2:** Distribution of background and obstetric characteristics of community-level factors, analysis from the 2019 EDHS, (weighted *N* = 1457)

Community-level variables	Minimum acceptable diet		Total (weighted), N (%)
	Acceptable, N (%)	Not acceptable, N (%)	
**Community education**			
Lower	85 (5.3)	895 (61.43)	980 (67.26)
Higher	79 (5.42)	398 (27.32)	477 (32.74)
**Residence**			
Urban	62 (4.26)	351 (24.9)	413 (28.34)
Rural	102 (7.00)	942 (64.65)	1044 (71.65)
**Region**			
Agrarian	147 (10.09)	1140 (78.24)	1287 (88.33)
Pastoralist	2 (0.14)	107 (7.34)	109 (7.48)
City	16 (1.10)	45 (3.09)	61 (4.19)
**Community poverty**			
Poor	49 (3.36)	503 (34.52)	552 (37.89)
Rich	116 (7.96)	789 (54.15)	905 (62.11)
**Community ANC utilization**			
lower	75 (5.15)	723 (49.62)	798 (54.77)
Higher	89 (6.11)	569 (39.05)	658 (45.16)
**Community Media access**			
Lower	102 (7.00)	988 (67.81)	1090 (74.81)
Higher	62 (4.26)	305 (20.93)	367 (25.19)

### Trends of dietary intake among children of age group 6–23 months in Ethiopia

**Fig. 2:**
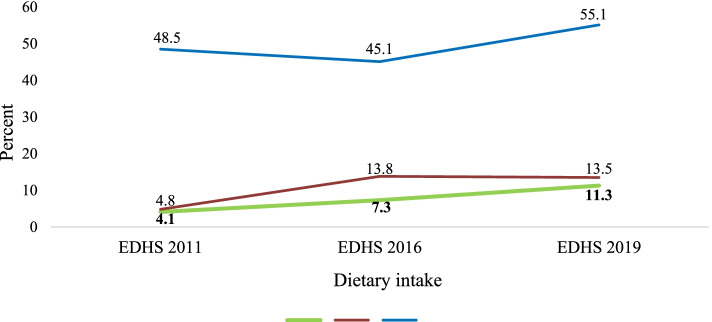
Trends of minimum dietary diversity, minimum meal frequency, and minimum acceptable diet among children of 6–23 months in Ethiopia from 2011, 2016, and 2019 EDHS data

The proportions of children aged 6–23 months who received the minimum acceptable diet in Ethiopia were 4.1% [95% C.I 3.4, 4.8], 7.3% [95% C.I 6.4, 8.2], and 11.3% [95% C.I 9.7, 12.9] during the survey periods of 2011, 2016, and 2019, respectively. As Fig. 2 shows, the trend of minimum dietary diversity significantly increased by 9% from 2011 to 2016. However, it declined from 2016 to 2019. The trends of minimum meal frequency decreased by 3.4% from the survey periods from 2011 to 2016, then significantly increased by 10% from the survey period of 2016 to 2019. The findings showed that the minimum acceptable diet increased by 3.3% during the survey period of 2011 to 2016 and by 4.0% during the survey period from 2016 to 2019 (Fig. 2).

### Factors influencing minimum acceptable diet among 6–23 months children in Ethiopia

After controlling for potential covariates a multi-level logistic regression model revealed that sociodemographic and economic variables (mothers’ education, child age, wealth index and residence), health services related factors (baby post-natal check-ups,) and access to media were statistically significant factors influencing the minimum acceptable diet among 6–23 months children in Ethiopia.

Infants and young children of mothers who attended primary education were about two times [aOR = 2.33 (95% C.I 1.25 to 4.35)], those who attended secondary education were three times [aOR = 2.49 (95% C.I 1.03 to 6.45)], and higher education were four times [aOR = 4.02 (95% C.I 1.53 to 10.54)] more likely to receive the recommended minimum acceptable diet compared to mother’s who never attended formal education.

Infants and young children of households who were in the medium and higher wealth status were about four times [aOR = 4.06 (95% C.I 1.41 to 11.72)], and five times [aOR = 4.91 (95% C.I 1.49 to 16.13)] more likely to receive the recommended minimum acceptable diet compared those in a lower wealth status respectively. The odds of receiving to receive the minimum acceptable diet were two times [aOR = 2.12 (95% C.I 1.25 to 3.58)] and [aOR = 2.23 (1.29 to 3.82)] among children of age group 12–18 months and 18–23 months, respectively compared to those in the age range of 6- 11 months. Findings also showed that the likelihood of receiving the minimum acceptable diet were two times aOR = 2.16 (95% C.I 1.31 to 3.55)] among children who received a postnatal check within 2 months of birth compared to their counterparts. Residency also predicted a minimum acceptable diet for infants and young children. The odds of receiving the recommended minimum acceptable diet were 3.4 times [aOR = 3.40 (95% C.I 1.73 to 6.68)] among urban residents compared to those who reside in the rural areas. Findings also showed that the likelihood of getting the minimum acceptable diet were 1.8 times [aOR 1.80 (95% C.1.17 to 2.77)] more likely among communities who had higher media exposure compared to their counterparts (Table 3).

**Table 3:** A multi-level fixed-effects logistic regression model showing factors influencing minimum acceptable diet among 6–23 months children in Ethiopia, 2019

Variable	Category	Model null	Model I AOR (95% CI)	Model II AOR (95% CI)	Model III AOR (95% CI)	***P***-value
Mothers’ educational status	No formal education		1.0		1.0	
	Primary		2.87 (1.66, 4.97) **		2.33 (1.25, 4.35) *	.008
	Secondary		3.47 (1.72, 7.03) *		2.49 (1.03, 6.45)*	.043
	Higher		7.08 (3.37, 14.87) **		4.02 (1.53, 10.54) *	.005
Household wealth index	Lowest		1.0		1.0	
	Medium		3.15 (1.51, 6.54) *		4.06 (1.41, 11.72) *	.010
	Highest		3.91 (1.86, 8.24) **		4.91 (1.49, 16.13) *	.009
Place of delivery	Home		1.0		1.0	
	Institutional		2.15 (1.22, 3.81) *		1.51 (.83, 2.75)	.175
Child age	6- 11 months		1.0		1.0	
	12–18 months		2.18 (1.29, 3.69) *		2.12 ( 1.25, 3.58)*	.006
	18–23 months		2.32 (1.36, 3.97) *		2.23 (1.29, 3.82) *	.004
Postnatal check within 2 months	Yes		2.13 (1.30, 3.49) *		2.16 (1.31, 3.55) *	.002
	No		1.0		1.0	
Community-women education	Higher			2.06 (1.31, 3.24) *	1.01 (.54, 1.89)	.208
	Lower			1.0	1.0	
Community poverty	Higher			3.10 (1.67, 5.78) *	.89 (.36, 2.25)	.180
	Lower			1.0	1.0	
Residence	Urban			4.15 (2.16, 7.96) **	3.40 (1.73, 6.68) **	.000
	Rural			1.0	1.0	
Region	Agrarian			3.64 (1.26, 10.51) *	2.74 (.93, 8.09)+	.068
	City			2.63 (.86, 8.07)	1.79 (.57, 5.57)	.317
	Pastoralists			1.0	1.0	
Community media exposure	Higher			1.93 (1.26, 2.95) **	1.80 (1.17, 2.77) *	.007
	Lower			1.0	1.0	
ICC (%)		23.3	21.6	22.6	19.9	
PCV (%)		Reference	95.2	97.9	90.6	
Model fitness Log likelihood		− 442.27	− 386.46	− 393.28	− 373.96	

*ICC* Intraclass correlation, *PCV* proportional changes in variance

* *p*-value <.05, ** *p*-value < 0.001, + *p*-value < 0.1

## Discussion

Understanding the trends and factors influencing the minimum acceptable at national level could provide an insight for designing contextually relevant strategies and policies. In this study, we found that 13.5, 55.1, and 11.3% of infants and young children aged 6–23 months in Ethiopia received minimum dietary diversity, minimum meal frequency, and minimum acceptable diet, respectively.

In the current study we found that nearly one out of eight (13.5%) infants and young children aged 6–23 months fed minimum dietary diversity in Ethiopia. This finding was nearly similar with study conducted in Sinan district, Southern Ethiopia, 13% [30]. However, it was by far lower than studies conducted in Debre Tabor town, Northern Ethiopia, 58.2% [31], Addis Ababa, 59.9% [32], in Bench Maji Zone, 38% [33] and Gorche district Southern Ethiopia, 29.9% [34], and Bale zone, Southern Oromia region 28.5% [35]. These discrepancy might be due to small sample size and urban setting in the previous studies. Thus, there is a need for behavioral change communication interventions especially in rural areas of Ethiopia such as awareness creation campaigns among communities especially among women of reproductive age groups. Indeed, empowering women is highly needed for improving the dietary diversity of the infants and young children [36]. Moreover, the trends of minimum dietary diversity showed an increment by 9% from 2011(4.8%) to 2016(13.8%). However, it was decreased by 0.3% from 2016(13.8) to 2019(13.5%). This implies infants and young children aged 6–23 months in Ethiopia have not been getting a diversified diet and are not in a good progress.

Regarding meal frequency this study found that more than half (55.1%) of infant and young children aged 6–23 months in Ethiopia received the recommended minimum meal frequency. This finding was lower than study studies conducted in Debre Tabor Town (76%) [31], Dabat District (72.2%) [37], Wolaita Sodo town (68.9%) [38], Bale Zone (68.4%) [35]. However, the finding was slightly higher than a study from Afar Region (43.8%) [22] and Dangila district (50.4%) [39]. The discrepancy might be due to unequal attention given to the infant and young child feeding by regions. The difference might be also due to pre-existing underlying factors such as drought and political instability. This suggests the need for strong commitment by the government and public health officials to work on infants and young children feeding. The finding also implies the importance of counselling mothers on child feeding practices, and provision of training on infant and child feeding to health extension workers and frontline health care providers.

Achieving either the minimum dietary diversity or minimum meal frequency cannot solve infant and young child feeding practices. Thus, focusing on the achievement of both recommended minimum dietary diversity and minimum meal frequency can help to ensure the minimum acceptable diet needed for infants and young children and has paramount relevance in improving children’s nutrition. In this study, we found that the recommended minimum acceptable diet for infants and young children aged 6–23 months in Ethiopia was only 11.3% [6]. The findings also showed that the recommended minimum acceptable diet has increased by only 7.2% (4.1% in 2011 and 11.3% in 2019) over the last ten years. This implies that nearly 9 out of 10 infants and children aged 6–23 months do not receive the minimum acceptable diet which can be due to a lack of obtaining a diversified diet and/or meeting the daily recommended meal frequency. This finding was far lower than those of studies conducted in Sri Lanka (63.3%) [40], Kenya (48.5%) [41], Ghana (29.9%) [42] and Uganda (23.9%) [43]. This difference may be due to differences in health systems among countries. This may also be due to the differences in support obtained from non-governmental organizations among different countries. This finding of the current study was in line with studies conducted in different areas of Ethiopia such as from Dembecha (8.6%) [44], and Goncha district (12.6%) [45]. The current study was also similar to studies from Malawi (8.36%) [44] and Nigeria (7.3%) [45]. This finding suggests that infants and young children in many countries especially low- and middle-income countries do not receive the recommended minimum acceptable diet. There is a need for economic and non-economic interventions among communities to improve infant and child feeding practices.

In this study, the determinants of minimum acceptable diet were also identified. The findings of this study showed that the educational status of the mother was associated with the attainment of the recommended minimum acceptable diet in infants and young children. The odds of receiving the minimum acceptable diet were higher among mothers who attended primary school and above educational levels compared to those who had no formal education. This finding was supported by previous studies from South Kivu, Democratic Republic of Congo [46], Dembecha district, Northwest Ethiopia [47], and Goncha district, Northwest Ethiopia [48]. This may be due to the fact that those who attended education might have the chance to learn about diet and child feeding practices. This might also be due to those who attended education may have the chance of getting information about infant and child feeding practices from different information sources such as books, encyclopedias, magazines, databases, newspapers, library Catalogues, and the internet. This implies the need for maternal nutrition education, especially in countries like Ethiopia where most women are uneducated and live in rural areas. The current study also pointed out that infants and children of households with medium and highest wealth indexes were more likely to receive the minimum acceptable diet compared to those with the lowest wealth index. This finding was similar to studies conducted in South Kivu, Democratic Republic of Congo [46], Philippines [49], and Goncha district, north West Ethiopia [48]. This may be due to the fact that diversifying diet and frequent feeding relies on the income of the households to secure their diet, which may be a difficulty for low-income countries like Ethiopia. However, it is possible to get a diversified, feasible, and healthy diet at affordable costs using locally available foods [50, 51]. Child age had an association with the minimum acceptable diet. Findings of this study showed that children in the age range of 12–18 months and 18–23 months were more likely to receive the recommended minimum acceptable diet compared to children in the age range of 6- 11 months. This finding was supported by a previous study from Ethiopia [52]. This may be due to perceptions that the diversity and frequency of younger children need to be increased gradually over time until the infants’ gastrointestinal systems matured over time. In the current study, we found that post-natal care determined the minimum acceptable diet for infants and young children. This finding was supported by a study from South Kivu, Democratic Republic of Congo [46]. This may be due to the reason that nutrition counseling and infant feeding are among the components of post-natal care. Women who attend postnatal care have the chance to receive counselling on nutrition and infant feeding practices and as a result, give the daily recommended minimum acceptable diet for their infants/children. The current study also showed that residency had an association with the minimum acceptable diet of infants and young children. The likelihood of receiving the recommended minimum acceptable diet was higher among urban residents compared to those who reside in rural areas. This finding was in line with studies from South Kivu, the Democratic Republic of Congo [46], and Ethiopia [52]. This might be due to information access. People who live in urban areas have the chance to get information from different information sources such as health care providers, television, databases, and the internet. This might also be due to the fact that people who live in urban areas are more educated than those who live in rural areas and as a result may have the knowledge, motivated to practice healthy behaviors. Our study also identified that media exposure was the predictor of the recommended minimum acceptable diet for infants and young children. This finding was supported by studies conducted in Ethiopia [48, 52]. This was due to the fact that some basic health services such as infant and young children feeding practices were transmitted through both local and international medias. This implies the need for behavioral change communication interventions such as counselling on the benefits receiving diversified diet frequently especially during the first one thousand days of child birth.

This study has several strengths and limitations. One of the strengths was that the study was based on EDHS data with a nationally representative large sample size population that covers all regions and city administrators of the country. The DHS surveys are similar in design having standard variables that are comparable across settings. In addition, the method of analysis was multilevel to accommodate the hierarchical nature of the EDHS, which takes into account the nested nature of the DHS data, thus allowing for the clustering effect of the outcome variable to be examined. Despite the above strengths, the study has the following limitations: The data relies on self-reported information and may be influenced by recall bias. The analysis was conducted using potential predictor variables extracted from the EDHS data. But data could have been more useful to this study if some particular information such as beliefs, perceptions, attitudes, and cultural experiences had been collected. This was a cross-sectional survey and doesn’t make causal inferences. Hence there is a need for conducting the study using strong study designs.

## Conclusions

In this study, we found that the minimum acceptable diet of infants and young children in Ethiopia was low and steady over the last ten years. Findings showed that sociodemographic and socioeconomic factors such as maternal education, child age, household wealth; and health system-related factors such as maternal postnatal check-ups had a significant influence on infants and young children’s MAD feeding. Indeed, community-level factors such as place of residence, and media exposure affect the MAD of infants and young children in Ethiopia. This implies the need for behavioral change communication interventions such as awareness creation campaigns, mothers’ counseling on infant and child feeding practices. Empowering women to prepare a diversified diet from the locally available food and frequently feed their infants and children is highly needed. Thus, we recommend health care providers and health extension workers to work strongly towards the improvement of the daily recommended minimum acceptable diet for infants and children. We also advise media personnel to transmit information about infant and young children feeding practices.

## Data Availability

Data is available online and you can access it from www.measuredhs.com.

## Abbreviations

ANC: Antenatal care
DHS: Demographic health survey
EDHS: Ethiopian demographic health survey
ICC: Intraclass correlation coefficient
PCV: Proportional change in variance
SNNP: Southern nations, nationalities, and peoples
